# How Xylenol Orange and Ferrous Ammonium Sulphate Influence the Dosimetric Properties of PVA–GTA Fricke Gel Dosimeters: A Spectrophotometric Study

**DOI:** 10.3390/gels8040204

**Published:** 2022-03-23

**Authors:** Martina Scotti, Paolo Arosio, Elisa Brambilla, Salvatore Gallo, Cristina Lenardi, Silvia Locarno, Francesco Orsini, Emanuele Pignoli, Luca Pedicone, Ivan Veronese

**Affiliations:** 1 Dipartimento di Fisica “Aldo Pontremoli”, Università degli Studi di Milano, 20133 Milano, Italy; martinascottims@gmail.com (M.S.); paolo.arosio@unimi.it (P.A.); cristina.lenardi@unimi.it (C.L.); silvia.locarno@unimi.it (S.L.); francesco.orsini@unimi.it (F.O.); luca.pedicone10@gmail.com (L.P.); ivan.veronese@unimi.it (I.V.); 2 Istituto Nazionale di Fisica Nucleare (INFN), Sezione di Milano, 20126 Milano, Italy; 3 Consorzio Interuniversitario Nazionale per la Scienza e Tecnologia dei Materiali (INSTM), 50121 Firenze, Italy; 4 Dipartimento di Scienze Farmaceutiche, Sezione di Chimica Generale e Organica “A. Marchesini”, Università degli Studi di Milano, 20133 Milano, Italy; elisa.brambilla@unimi.it; 5 Fondazione IRCCS “Istituto Nazionale dei Tumori”, 20133 Milano, Italy; emanuele.pignoli@unimi.it

**Keywords:** Fricke gel dosimetry, xylenol orange sodium salt, ferrous ammonium sulphate, PVA-GTA hydrogel

## Abstract

The development of Fricke gel (FG) dosimeters based on poly(vinyl alcohol) (PVA) as the gelling agent and glutaraldehyde (GTA) as the cross-linker has enabled significant improvements in the dose response and the stability over time of spatial radiation dose distributions. However, a standard procedure for preparing FG in terms of reagent concentrations is still missing in the literature. This study aims to investigate, by means of spectrophotometric analyses, how the sensitivity to the radiation dose and the range of linearity of the dose–response curve of PVA-GTA-FG dosimeters loaded with xylenol orange sodium salt (XO) are influenced by ferrous ammonium sulphate (FAS) and XO concentrations. Moreover, the effect of different concentrations of such compounds on self-oxidation phenomena in the dosimeters was evaluated. PVA-GTA-FG dosimeters were prepared using XO concentrations in the range 0.04–0.80 mM and FAS in the range 0.05–5.00 mM. The optical absorbance properties and the dose response of FG were investigated in the interval 0.0–42.0 Gy. The results demonstrate that the amount of FAS and XO determines both the sensitivity to the absorbed dose and the interval of linearity of the dose–response curve. The study suggests that the best performances of FG dosimeters for spectrophotometric analyses can be obtained using 1.00–0.40 mM and 0.200–0.166 mM concentrations of FAS and XO, respectively.

## 1. Introduction

Fricke gel (FG) dosimeters are chemical dosimeters prepared by infusing a ferrous ammonium sulphate (FAS) solution (i.e., the Fricke solution [1]) into a hydrogel matrix. The interaction of ionizing radiation with the molecules of the hydrogel and the consequent formation of free radicals activate different chemical routes that lead to the oxidation of ferrous ions (Fe^2+^). The final concentration of radiation-induced Fe^3+^ ions is proportional to the energy deposited by ionizing radiation in the dosimeter, i.e., the absorbed dose. Three-dimensional (3D) spatial information on the absorbed dose is obtainable within the gel volume, and it can be captured and retrieved by a suitable readout technique [2]. Magnetic Resonance Imaging (MRI) is the main imaging modality of FG dosimeters and relies on the dose-dependent changes in nuclear relaxation times of the hydrogen nuclear spins caused by radiation exposure and consequent iron oxidation. Indeed, Fe^2+^ and Fe^3+^ ions have different paramagnetic features and perturb the relaxation times of neighboring water protons differently [2,3,4].

Alternatively, Optical Computed Tomography (OCT) can be used to quantify localized variations in the optical attenuation coefficient, which are proportional to the absorbed dose. In fact, in FG dosimeters, spectrophotometric determination of the light absorption and hence concentration of ferric ions is achievable using visible (Vis) light when using a suitable ligand that moves the absorption bands of Fe^3+^ from ultraviolet (UV) to longer wavelengths. One of the most used chelating agents is xylenol orange sodium salt (XO) [5]; when added to the Fricke solution, XO chelates Fe^3+^ ions, creating chemical species characterized by a broad absorption band that peaks at around 585 nm (further explanation of the possible Fe^3+^–XO complexes and their optical absorptions is given in the Section 3). Furthermore, XO reduces the diffusion of ferric ions within the gel matrix and hence the loss of dose localization that is a known limitation of FG dosimeters [2,3,4].

The Fricke solution underlying FG dosimeters is a well-established chemical dosimeter in liquid form (i.e., the Fricke dosimeter), and it is also used as a primary standard for absorbing the dose in water in various ionizing radiation metrology laboratories. In fact, the composition of the Fricke dosimeter in terms of chemical species and concentration is standardized. Similarly, the chemical yield of ferric ions *G*(Fe^3+^) in Fricke solution was obtained with high accuracy by the comparison of the Fricke dosimeter with calorimetric standards [2].

By contrast, a lack of harmonization in the composition of FG dosimeters emerges from the literature. This is essentially a consequence of the fact that FG dosimeters are still a subject of research in various laboratories that are trying to overcome the current limits of these dosimeters, which are mainly related to their poor temporal stability due to self-oxidation and Fe^3+^ diffusion phenomena. In fact, attempts to improve the dosimetric properties of FG dosimeters resulted in several studies on different chemical formulations obtained by using further organic additives such as saccharides [6,7,8], antioxidants [9,10,11,12], nanocomposites [13,14,15], chelating agent alternatives to xylenol orange [16,17,18,19,20,21,22,23], and, finally, different gel matrices acting as mobility-reducing agents [24,25,26,27,28].

A non-exhaustive overview of several compositions of xylenol orange–FG dosimeters investigated by different research groups is given in Table 1.

Considering the gel matrices used to prepare FG dosimeters, the interest in hydrogels obtained with poly(vinyl alcohol) (PVA) and cross-linked by glutaraldehyde (GTA) is increasing [19,20,25,26,35,58,59,60,61]. Indeed, compared with natural gelling agents such as gelatin and agarose, such synthetic compounds allow for higher levels of reproducibility in the manufacturing process of FG dosimeters and slower diffusion of Fe^3+^ ions within the gel matrix [38,39,58].

As already observed in natural-matrix-based FG dosimeters [47], and also in PVA-GTA-FG dosimeters, the dosimetric properties are expected to be influenced by the concentrations of FAS and chelating agent used to prepare the dosimeter. However, to the best of the authors’ knowledge, no systematic studies on such dependences are available in the literature for these types of FG dosimeters. Therefore, this study aims to investigate, by means of spectrophotometric analyses, how the sensitivity to the radiation dose and the range of linearity of the dose–response curve of PVA-GTA-FG loaded with XO (XO-PVA-GTA-FG) dosimeters are influenced by FAS and XO concentrations. In parallel, the effect of different concentrations of such compounds on self-oxidation phenomena occurring in the investigated FG dosimeters was evaluated.

## 2. Materials and Methods

The procedure used for the preparation of XO-PVA-GTA-FG dosimeters is well-established and has been described in previous papers [58]. All batches of FG dosimeters were prepared using ultrapure water obtained by a water purification system (Milli-Q^®^ Direct, EMD Millipore, Burlington, VT, USA) and analytical-grade reagents. In this study, twenty-one distinct sets of samples, characterized by different concentrations of ferrous ammonium sulphate hexahydrate (FAS, Carlo Erba, Val-de-Reuil, FR) and xylenol orange tetra-sodium salt (XO, Sigma-Aldrich, Saint Louis, MO, USA), were prepared. Details of the XO and FAS concentrations in the samples are given in Table 2. The use of such concentrations enabled us to cover an [FAS]/[XO] ratio from 0.25 to 25.0.

The final concentration of the remaining reagents employed for the preparation of dosimeters was equal to 8.7% (*w*/*w*) for poly(vinyl alcohol) (PVA, Mowiol^®^-20-88, M_w_ ~125 kDa, Sigma-Aldrich), 27.7 mM for glutaraldehyde (GTA, Sigma-Aldrich), and 27.0 mM for sulfuric acid (Sigma-Aldrich).

For each set, at least 25 dosimeters inside 10 mm optical path length poly(methyl-methacrylate) (PMMA) cuvettes were obtained.

After the complete gelation, all FG dosimeters were sealed, protected from light, kept refrigerated at the controlled temperature of 6 °C for 1 day, and brought back to room temperature 1 h before the irradiations and the spectrophotometric measurements.

The samples were irradiated with an IBL 437C ^137^Cs blood irradiator at the “Fondazione IRCCS Istituto Nazionale dei Tumori” of Milano, Italy at room temperature using a dose rate of 11 cGy/s. Dose intervals of 0–36 Gy and 0–42 Gy were used for the samples of Sets 1–6 and 7–21, respectively. Three dosimeters of each set were irradiated for each dose value. Optical absorbance (OA) measurements of un-irradiated and irradiated samples were carried out with a UV–Vis spectrophotometer (Cary 100 UV–Vis, Agilent Technologies, Santa Clara, CA, USA) in the wavelength range of 360–720 nm with steps of 1 nm. OA spectra were acquired using one cuvette filled with ultrapure water as a reference.

Furthermore, in order to investigate self-oxidation phenomena, three un-irradiated samples of the Sets 1–15 of Table 2 were placed inside a thermostatic bath at the temperature of 21.0 ± 0.5 °C. After a thermalization time of 15 min, OA spectra of these samples were measured at regular times t_i_, starting from t_0_ = 0 up to t_f_ = 90 min, in approximately 13-min steps.

## 3. Results and Discussion

### 3.1. FAS Variation

In FG dosimetry, OA spectra of each sample are generally reported as differences (ΔOA) between the OA spectrum measured after and before the exposure to ionizing radiation. Indeed, the quantity ΔOA evaluated at a suitable wavelength or in a suitable wavelength range can be directly correlated to the absorbed dose. When XO is used as the chelating agent in FG dosimeters, negative values of ΔOA are expected in a wavelength region centered at around 430 nm where the absorption band of free XO occurs. Indeed, the increase in the concentration of Fe^3+^ ions, while increasing the radiation dose, gave rise to a decrease in XO molecules not bounded with ferric ions.

**Figure 1:**
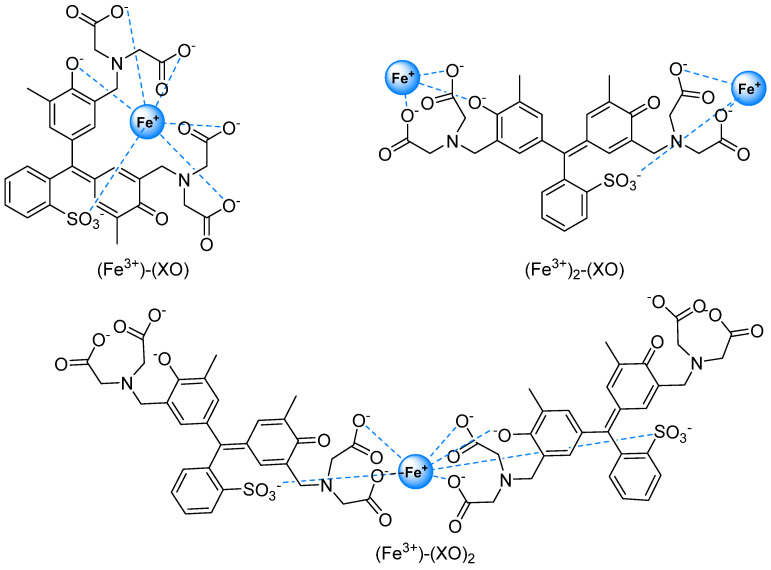
Xylenol orange–iron complexes.

By contrast, positive values of ΔOA in a broad wavelength interval at around 500–650 nm can be detected and correspond to partially overlapping absorption bands due to various ferric ions and xylenol orange complexes [62,63]. In fact, XO is able to bind one or two metal ions at both of its ends in a π-electron conjugated system thanks to the presence of the iminodiacetic acid groups linked to the chromophoric moiety, as well as by phenolate oxygen atoms. The most representative complexes present three different stoichiometric ratios between XO and ferric ions: (Fe^3+^)-(XO)_2_, (Fe^3+^)-(XO), and (Fe^3+^)_2_-(XO) (Figure 1) [63,64].

The probability of each complex’s formation depends on the Fe^3+^ and XO concentrations [62]. For example, it is known from the literature [46,62] that increasing the concentration of Fe^3+^ ions or XO favors the formation of the (Fe^3+^)_2_-(XO) complex or the (Fe^3+^)-(XO)_2_ complex, respectively. Upon Fe^3+^ binding, the yellow-orange color of FG dosimeters loaded with XO changes to violet, allowing us to point out the formation of the complex in the visible range. In fact, the (Fe^3+^)_2_-(XO) and (Fe^3+^)-(XO) complexes present an absorption band in the range of approximatively 500–620 nm, while the (Fe^3+^)-(XO)_2_ complex absorbs light at a shorter wavelength in the spectral region overlapping the tail of the main absorption peak of the free XO at 430 nm [63].

**Figure 2:**
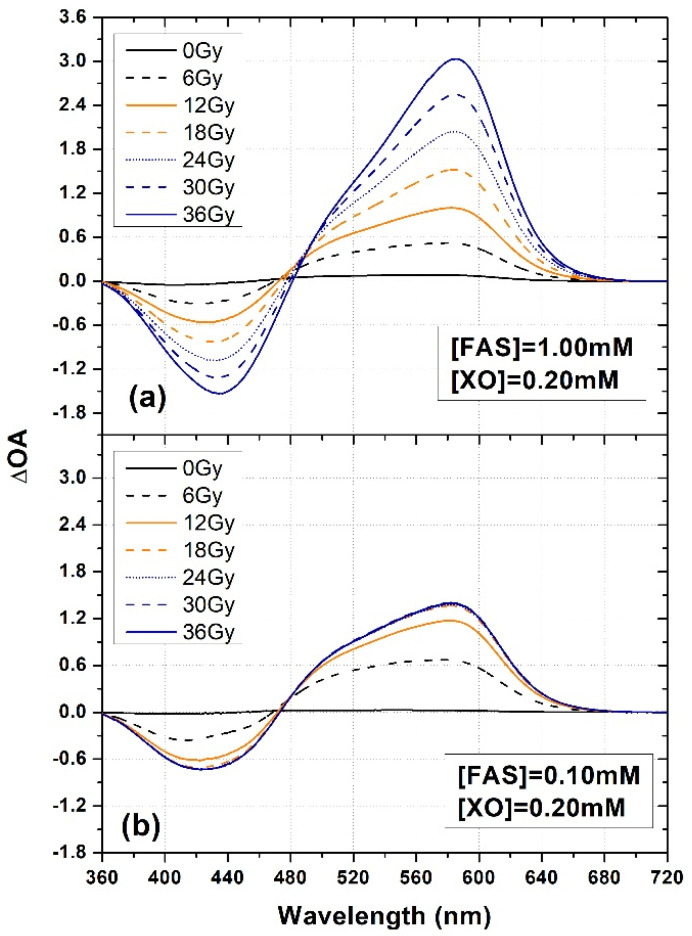
Examples of ΔOA spectra of XO-PVA-GTA-FG dosimeters prepared with (XO) = 0.20 mM and using two different FAS concentrations: (**a**) (FAS) = 1.00 mM and (**b**) (FAS) = 0.20 mM. The samples were irradiated at various doses.

Examples of ΔOA spectra of XO-PVA-GTA-FG dosimeters prepared with an XO concentration of 0.200 mM and two different FAS concentrations (equal to 0.10 mM and 1.00 mM, i.e., Sets 2 and 5 of Table 2, respectively), irradiated to various doses, are shown in Figure 2. A saturation effect can be clearly observed for the dosimeters prepared with an FAS concentration of 0.10 mM (Figure 2b). In fact, the ΔOA spectra related to doses above 12 Gy were fully overlapping, indicating the full depletion of Fe^2+^ in the dosimeters.

**Figure 3:**
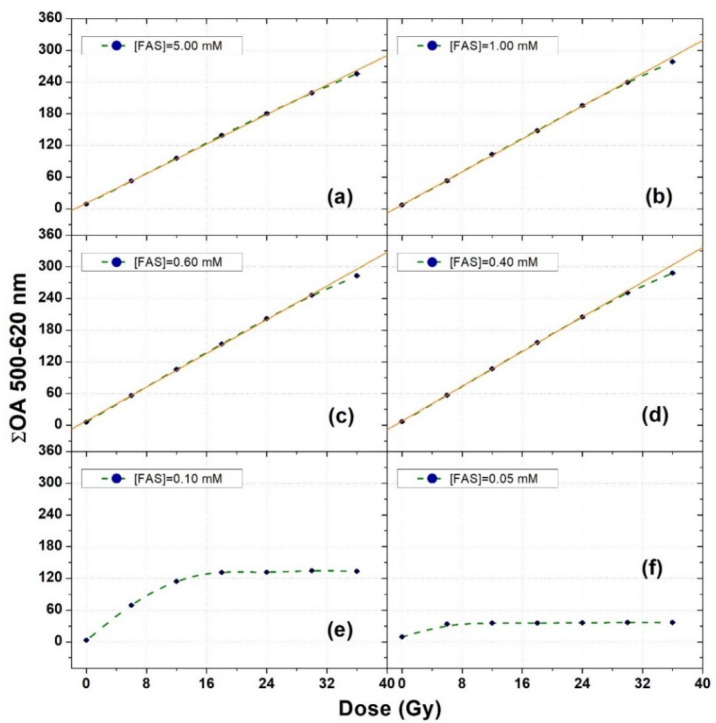
Dose–response curves of XO-PVA-GTA-FG dosimeters prepared using different FAS concentrations at a fixed XO concentration of 0.20 mM. The orange straight lines are the linear fits to the experimental data. The dashed green lines were drawn to guide the eyes. The error bars correspond to one standard deviation and are smaller than the symbol dimensions.

By considering the whole set of dosimeters prepared with different FAS concentrations (i.e., Sets 1–6 of Table 2) irradiated at different doses, and integrating their ΔOA spectra in the wavelength interval (500–620 nm), the dose–response curves shown in Figure 3 were obtained. Each data point of Figure 3 corresponds to the average over three different samples. The error bars (one standard deviation) are smaller than the dimensions of the symbols.

For the samples with FAS concentrations equal to 0.40 mM, 0.60 mM, 1.00 mM, and 5.00 mM, straight lines were fitted to the experimental data in the dose interval 0–30 Gy (solid orange lines in Figure 3). The results of the fit parameters are given in Table 3. For the remaining samples with FAS concentrations of 0.10 mM and 0.05 mM, no fits were performed because of the limited number of data points showing a dynamic trend of the dosimeter response with the radiation dose.

The slope values of Table 3 indicate that a slight decrease in the sensitivity of the dosimeters with increasing the FAS concentration from 0.40 mM to 5.00 mM occurred in the investigated XO-PVA-GTA-FG dosimeters. In addition, such a slight decrease in the sensitivity was associated with a better linearity above 30 Gy. However, it is worth noting that a satisfactory linear dose response up to at least 30 Gy was observed in all the FG dosimeters with an FAS concentration ranging from 0.40 mM to 5.00 mM.

These findings confirm that, for a fixed XO concentration of 0.200 mM, there is a rather wide range of FAS concentrations that can be employed for the preparation of XO-PVA-GTA-FG dosimeters without expecting significant changes in their main dosimetric features. Actually, most of the research available in the literature about XO-FG dosimeters was performed using FAS concentrations in the interval 0.50–1.50 mM (i.e., an [FAS]/[XO] ratio from 1 to 10), independently of the employed gelling matrix (see Table 1).

### 3.2. XO Variation

**Figure 4:**
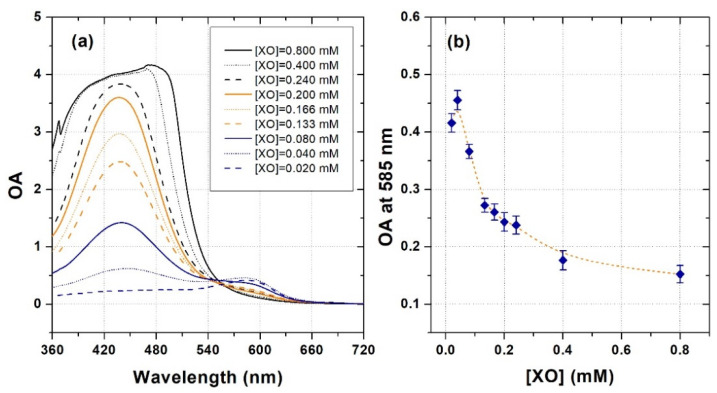
(**a**) Examples of optical absorbance spectra of un-irradiated XO-PVA-GTA-FG dosimeters prepared using different XO concentrations at a fixed FAS concentration of 0.40 mM. One cuvette filled with ultrapure water as a reference. (**b**) Trend of the optical absorbance at 585 nm vs. XO concentration. The dashed orange line was drawn to guide the eyes. The error bars correspond to one standard deviation.

Figure 4a shows the OA spectra of un-irradiated XO-PVA-GTA-FG dosimeters prepared with a FAS concentration of 0.40 mM and different XO concentrations ranging from 0.020 mM to 0.800 mM (i.e., Sets 7–15 of Table 2). As expected, the presence of XO molecules in the dosimeters gave rise to a broad main absorption band centered at approximately 430 nm [63]. The amplitude of this peak increased as the XO concentration increased and for the samples prepared with XO concentrations of 0.400 mM and 0.800 mM instrumental saturation occurred.

An absorption band centered at approximately 585 nm can be also observed in the OA spectra of the dosimeters prepared with very low XO concentrations (i.e., ≤0.080 mM). This peak can be explained by the presence of Fe^3+^ ions produced by self-oxidation phenomena and the formation of (Fe^3+^)_2_-(XO) and (Fe^3+^)-(XO) complexes. Indeed, such complexes are characterized by a main OA peak at 585 nm [63]. For higher XO concentrations, Fe^3+^-(XO)_2_ complexes are expected to be the major species. Such complexes absorb light at a shorter wavelength [63], i.e., in the spectral region overlapping the tail of the main absorption peak of the free XO at 430 nm. The complete trend of the OA at 585 nm vs. XO concentration is shown in Figure 4b.

**Figure 5:**
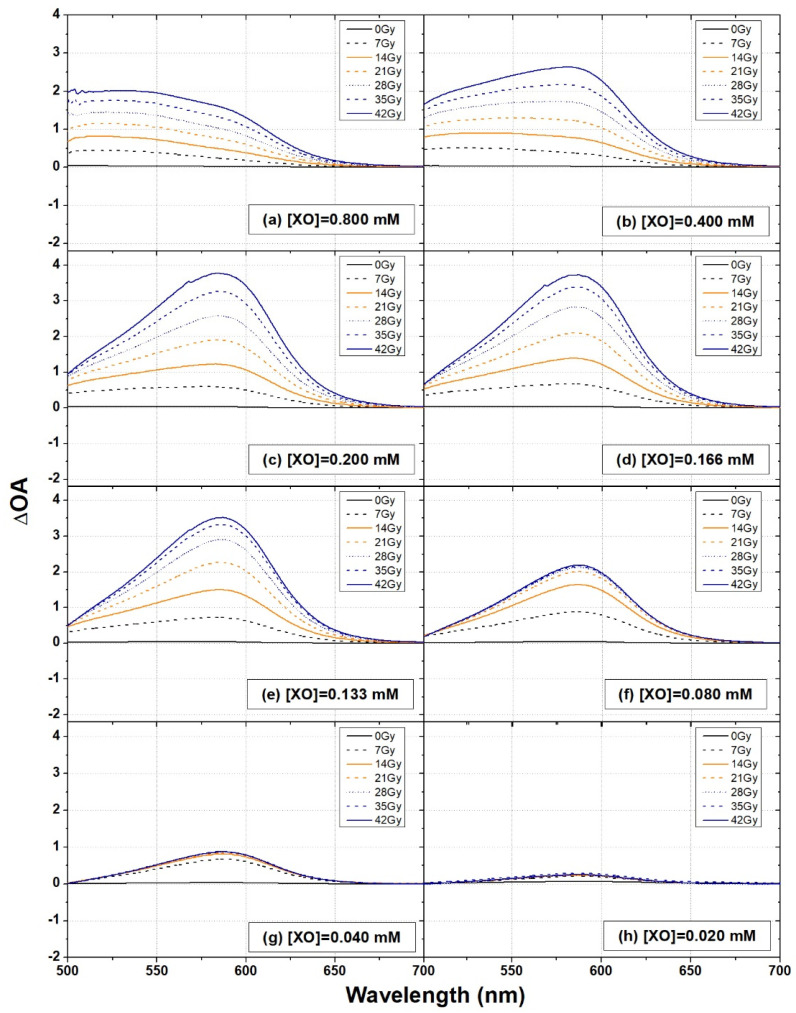
Examples of ΔOA spectra of XO-PVA-GTA-FG dosimeters prepared using different XO concentrations and irradiated at various doses.

Examples of ΔOA spectra of XO-PVA-GTA-FG dosimeters prepared with an FAS concentration of 0.40 mM and different XO concentrations and irradiated at various doses are shown in Figure 5.

Only the spectral region of interest for dosimetric purposes (i.e., the wavelength interval where the absorption bands related to XO–Fe complexes occurred) was considered. It is worth noting that the boundary of the absorption region strictly depends on the XO concentration. In fact, the shape of the ΔOA spectra of the dosimeters prepared with the highest XO concentration of 0.800 mM (Figure 5a) was different from those measured in the dosimeters with an XO concentration lower than 0.400 mM (Figure 5c–h), independently of the dose. Indeed, the highest ΔOA values in Figure 5a occurred at a wavelength lower than 585 nm. This could be explained by considering that, when increasing the XO concentration, the formation of the complex 1:2 (Fe^3+^)-(XO)_2_ is predominant, presenting an absorption peak under 500 nm. When decreasing the concentration, the main complex becomes the 1:1 (Fe^3+^)-(XO) complex with a peak at about 585 nm in an acidic medium. However, the complex (Fe^3+^)_2_-(XO), prevailing when the iron concentration is higher than the XO concentration, also shows an absorption peak at the same wavelength [62,63]. Thus, the exact attribution of the maximum optical absorption is difficult because the mentioned complexes are present in equilibrium in the solution.

Actually, the shape of the ΔOA spectra of Figure 5a suggests that the (Fe^3+^)-(XO)_2_ complexes make a greater contribution than the other ones due to the effect of the availability of XO molecules that can be bounded with radiation-induced ferric ions. Consequently, in the samples with the highest XO concentration the absorption band related to (Fe^3+^)_2_-(XO) and (Fe^3+^)-(XO) that peaked at 585 nm appeared to only be a shoulder of the main absorption band that peaked at a lower wavelength and was related to the (Fe^3+^)-(XO)_2_ complexes [62].

A similar shape was observed for the ΔOA spectra of the samples prepared with a XO concentration of 0.400 mM, but only for doses ≤14 Gy (Figure 5b).

Actually, the relative ratio between the concentration of xylenol orange and the concentration of ferric ions in complexes with different stoichiometric ratios (and consequently their absorption bands) depends on the dose, i.e., on the concentration of ferric ions produced in the dosimeters after exposure to ionizing radiation [14,59].

The samples with XO concentrations of 0.200 mM and 0.166 mM (Figure 5c,d) were characterized by the well-known ΔOA spectra, such as the one described in Figure 2a, and showed a systematic increase in their intensity as the radiation dose increased. For lower XO concentrations (Figure 5e–h), the dynamic trend with the radiation dose was progressively lost and, for the lowest XO concentration of 0.020 mM, the ΔOA spectra fully overlapped each other.

The observed saturation effects of the response of these dosimeters were attributable to the low concentration of XO molecules that can be bounded with the radiation-induced ferric ions.

**Figure 6:**
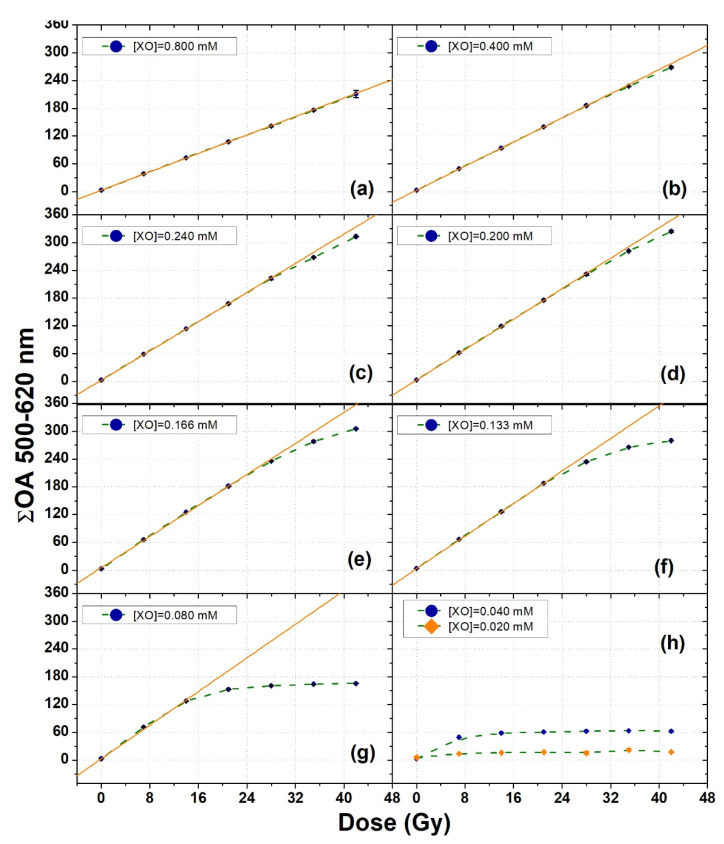
Dose–response curves of XO-PVA-GTA-FG dosimeters prepared using different XO concentrations at a 0.40 mM concentration of FAS. The orange straight lines are the linear fits to the experimental data. The dashed green lines were drawn to guide the eyes. The error bars correspond to one standard deviation and are smaller than the symbol dimensions.

A thorough analysis of the dose–response curve of the XO-PVA-GTA-FG dosimeters prepared with an FAS concentration of 0.40 mM and different XO concentrations is shown in Figure 6, where the cumulative values of ΔOA in the spectral interval 500 nm–620 nm (ΣOA) were plotted versus radiation dose. Each data point of Figure 6 corresponds to the average over three different samples.

For the samples with an XO concentration ranging from 0.080 mM to 0.800 mM, straight lines were fitted to the experimental data. The results of the fit parameters, together with details of the dose interval considered for the fitting procedure, are given in Table 4. For the remaining samples with XO concentrations of 0.020 mM and 0.040 mM, no fits were performed because of the limited number of data points showing a dynamic trend with the radiation dose.

The slope values of Table 4 demonstrate a systematic increase in the sensitivity of the dosimeters with a decrease in the XO concentration. Such an increase was associated with a contraction of the interval where the dose–response curve proved to be linear.

**Figure 7:**
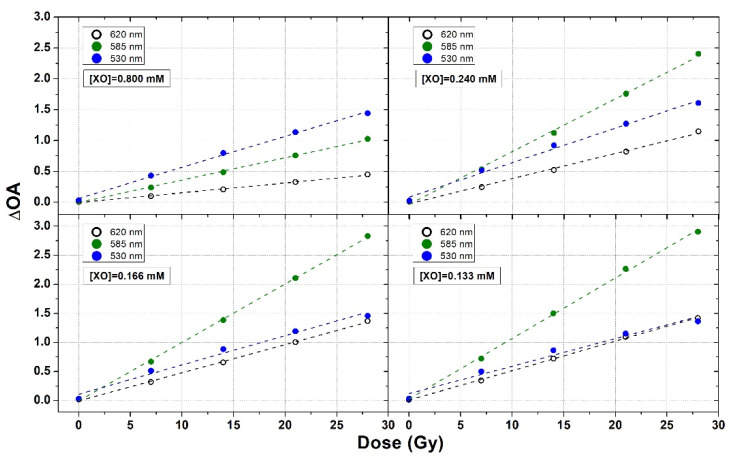
Examples of ΔOA at 620, 585, and 530 nm of PVA-GTA-FG dosimeters irradiated at various doses in the interval 0.0–28.0 Gy and prepared using different XO concentrations.

In addition to the use of the cumulative ΔOA, dose–response curves similar to those of Figure 6 were obtained by considering the ΔOA values calculated at individual wavelengths in the interval 500–630 nm in 5-nm steps. Several examples of such curves in dosimeters prepared with XO concentrations of 0.800, 0.240, 0.166, and 0.133 (i.e., Sets 15, 13, 11, and 10 of Table 2) related to the selected wavelengths of 630, 585, and 530 nm are shown in Figure 7.

A straight line was fitted to each dose–response curve and the sensitivity to the radiation dose (i.e., the slope of the fitted straight line) for each sample at each individual wavelength was accordingly obtained.

**Figure 8:**
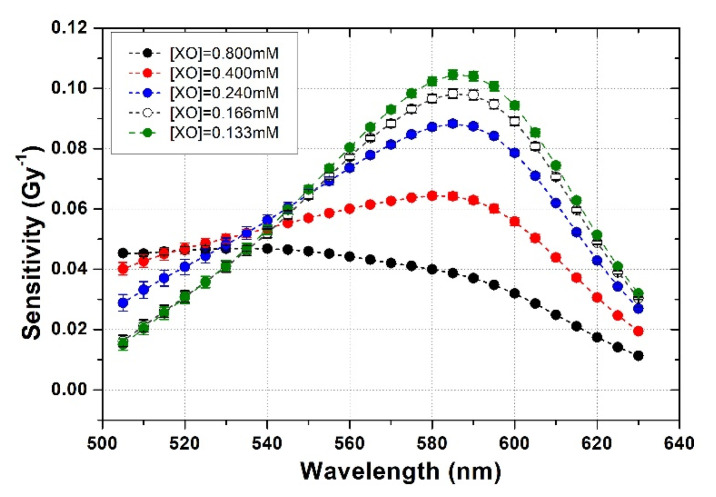
Slope of the fitted dose–response straight lines versus wavelength for some of the studied Fricke gel dosimeters.

The complete results of the wavelength-dependence of the sensitivity to the radiation dose for XO-PVA-GTA-FG dosimeters prepared with different XO concentrations are shown in Figure 8, where the slope values of the fitted straight lines vs. wavelength are plotted. The trend observed in Figure 8 confirmed the highest sensitivity at 585 nm for all the samples, except the ones prepared with the maximum XO concentration of 0.800 mM.

### 3.3. Fine Tuning of FAS and XO Concentrations

**Figure 9:**
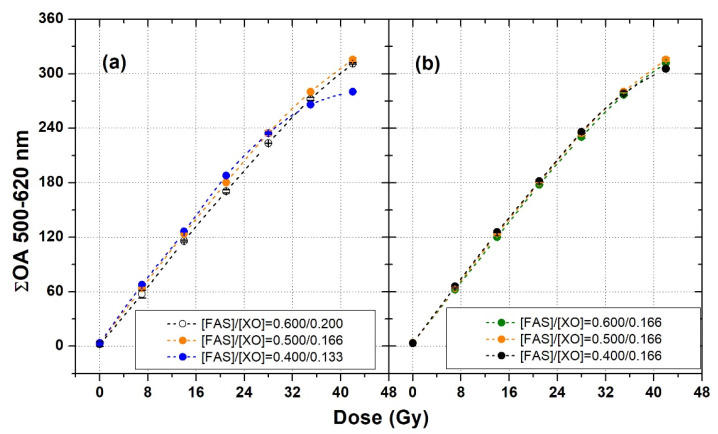
Dose–response curves of XO-PVA-GTA-FG dosimeters obtained by slight changes in FAS and XO concentrations. (**a**) [FAS]/[XO] ratio equal to 3; (**b**) (XO) = 0.166 mM and (FAS) = 0.6, 0.5, and 0.4 mM. The error bars correspond to one standard deviation and are smaller than the symbol dimensions. The dashed lines were drawn to guide the eyes.

The results of the analysis of the dose–response curves of XO-PVA-GTA-FG dosimeters prepared with different FAS and XO concentrations indicate that the use of an FAS concentration in the interval 0.40–0.60 mM, coupled with the use of an XO concentration in the interval 0.133–0.200 mM, guaranteed satisfactory dosimetric properties of the samples both in terms of sensitivity and linearity (Figure 9).

Figure 9a shows the dose–response curves of three different sets of samples (Sets 16–18 of Table 1) prepared by maintaining the [FAS]/[XO] concentration ratio equal to 3.0. The three curves were rather similar: For doses ≤35 Gy, the maximum variation among the cumulative ΔOA values of the samples was found to be equal to 8%. At higher doses, the saturation effect was more evident for the FAS concentration of 0.40 mM. A significantly lower variability was observed among XO-PVA-GTA-FG dosimeters prepared with a FAS concentration in the interval 0.40–0.60 mM but using a constant XO concentration of 0.166 mM (Sets 19–21 of Table 1).

The dose–response curves of these samples are plotted in Figure 9b. In this case, within the entire investigated dose interval, the maximum variation among the cumulative ΔOA values of the samples was assessed to be equal to 3.0%.

### 3.4. Self-Oxidation

**Figure 10:**
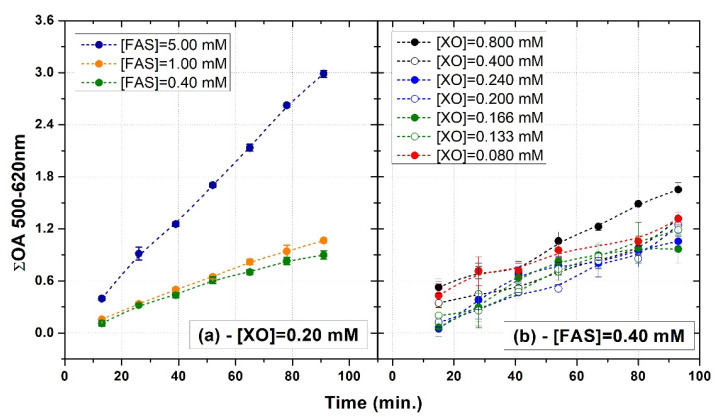
Examples of the change in the cumulative OA over time measured in un-irradiated XO-PVA-GTA-FG dosimeters prepared with different concentrations of FAS (**a**) and XO (**b**). The error bars correspond to one standard deviation. The dashed lines were drawn to guide the eyes.

Besides the optimization of FAS and XO concentrations to guarantee an adequate level of sensitivity and a wide range of linearity, the effects of such compounds on the self-oxidation features of XO-PVA-GTA-FG dosimeters were investigated. Figure 10a,b show examples of the change in the cumulative OA over time measured in un-irradiated XO-PVA-GTA-FG dosimeters prepared with different concentrations of FAS and XO, respectively. Each data point represents the difference between the cumulative OA measured at the time t_i_ and that obtained at the time t_0_.

The results suggest that the self-oxidation rate did not significantly depend on the XO concentration when an FAS concentration of 0.40 mM was used (Figure 10b). Similar self-oxidation trends were observed in samples with an XO concentration of 0.200 mM and FAS concentrations ranging from 0.40 mM to 1.00 mM. By contrast, XO-PVA-GTA-FG dosimeters prepared with an FAS concentration of 5.00 mM showed faster self-oxidation and after 60 min the cumulative OA was three times higher than the value of samples prepared with lower FAS concentrations.

## 4. Conclusions

A systematic study on the effects of variation in ferrous ammonium sulfate (FAS) and xylenol orange (XO) concentrations on the dosimetric properties of Fricke gel dosimeters prepared with poly(vinyl alcohol) (PVA) cross-linked by glutaraldehyde (GTA) was carried out. The investigated properties concerned the dose–response curves (i.e., the sensitivity and range of linearity), the self-oxidation rate, and the level of self-oxidation.

From the outcomes achieved in this study, some conclusions can be drawn about the behavior of the tested XO-PVA-GTA-FG dosimeters. Firstly, increasing the FAS concentration does not significantly increase the absorbed dose–optical response range, nor does it increase the optical sensitivity. However, a more pronounced level of self-oxidation was noticed; thus, an increase in the FAS concentration tends to decrease the temporal stability. On the other hand, lower FAS concentrations reduce the dosimeter’s response range. However, there was no evidence of variations for the optical sensitivity. Furthermore, it was found that the XO concentration is the main factor responsible for the limited absorbed dose response.

Starting from these considerations, the experimental data were in line with the literature data on traditional and natural gel matrices. In particular, 1.00–0.40 mM and 0.200–0.166 mM are the optimal intervals of FAS and XO concentrations, respectively, to be used in the preparation of dosimeters in order to maximize their performances in the case of spectrophotometric analyses.

The results obtained in this paper allow us to begin a new investigation on the possibility of improving the dosimetric stability of the FG by adding alternative chelating agents and/or antioxidants, such as sulfosalicylic acid (SSA), methylthymool blue sodium salt (MTB), ethylenediaminetetraacetic acid (EDTA), and dimethylsulfoxide (DMSO).

## Figures and Tables

**Table 1:** Composition of various Fricke gel dosimeters available in the literature prepared with different gel agent (GA), ferrous ammonium sulphate (FAS), and xylenol orange (XO) contents.

Year	Author	Gel Agent (GA)	GA (%)	FAS (mM)	XO (mM)
**2022**	Piotrowski et al. [29]	Pluronic F-127	25.0	0.01–5.00	0.03–0.50
**2021**	Dudek et al. [28]	Pluronic F-127	25.0	1.00	0.165
**2021**	Farajzadeh & Sina [30]	Gelatin	0–220 mM	0.02–2.50	0.02–0.20
**2021**	Pérez et al. [31]	Gelatin	3.0	1.0	0.165
**2021**	Gallo et al. [32]	PVA + GTA	8.0	0.5	0.165
**2019**	Smith et al. [33]	PVA	10.0–20.0	0.4	0.20–0.40
		Gelatin	10.0	0.1–0.4	0.10–0.40
**2019**	Vedelago et al. [34]	Gelatin	4.0	0.3–0.6	0.10–0.20
**2019**	Babu et al. [11]	Gelatin	5.0	0.3	0.050
**2019**	Lazzeri et al. [35]	PVA + GTA	10.0–12.5	0.5	0.165
**2018**	Lazzaroni et al. [36]	PVA + GTA	10.0	0.5	0.165
		Gelatin	3.0	0.5	0.165
**2017**	Welch et al. [37]	Gelatin	6.0	0.3	0.050
**2017**	Marini et al. [38]	PVA + GTA	9.1	0.5	0.165
		Gelatin	2.9	0.5	0.165
**2017**	Marrale et al. [39]	PVA + GTA	10.0	1.5	0.165
		Agarose	3.0	1.5	0.165
**2017**	Soliman et al. [40]	Gelatin	4.0	1.0	0.100
**2017**	Gambarini et al. [41]	Gelatin	3.0	1.0	0.165
		Agarose	1.5	1.0	0.165
**2017**	Del Lama et al. [42]	Gelatin	0–250 mM	0.3–5.0	0.05–0.25
**2016**	El Gohary et al. [43]	Gelatin	4.0	1.0	0.10
**2014**	Marrale et al. [44]	Agarose	3.0	0.5–5.0	0.165
**2010**	Cavinato et al. [45]	Gelatin	5.0	1.0	0.1
**2009**	Babic et al. [46]	Gelatin	6.0	1.0	0.05
**2008**	Babic et al. [47]	Gelatin	4.0	0.1–0.9	0.025–0.100
**2008**	Davies et al. [48]	Gelatin	3.85	1.0	0.10
**2008**	Galante et al. [49]	Gelatin	1.0, 5.0, 10.0	1.0	0.10
**2003**	Healy et al. [6]	Agarose	1.0	0.4	0.20
**2002**	Hill et al. [50]	PVA	20.0	0.4	0.40
**2000**	Chu et al. [24]	PVA	15.0, 20.0, 25.0	0.2–0.8	0.20–0.80
**1997**	Pedersen et al. [51]	Gelatin	4.0	1.5	1.50
		Agarose	1.5–3.0		
**1997**	Kron et al. [52]	Gelatin	2.0–10.0	0.5–1.0	0.02–025
		Agarose	1.0–1.5	0.25	
**1996**	Rae et al. [53]	Gelatin	4.0	0.2	0.20
**1996**	Tarte et al. [54]	Agarose	1.0	0.4	0.20
**1991**	Appleby et al. [55]	Agarose	1.5	0.4	0.04–0.06
**1987**	Appleby et al. [56]	Agarose	1.5	0.2	0.0
**1984**	Gore et al. [57]	Gelatin	4.0	1.0	0.0

**Table 2:** Details of XO and FAS concentrations and their ratio in the investigated set of samples. Sets 12, 16, and 19 had the same XO and FAS concentrations as Sets 3, 10, and 11, respectively, but they were prepared at different times and used in distinct experiments.

SET	XO (mM)	FAS (mM)	[FAS]/[XO] Ratio
**1**	0.200	0.05	0.25
**2**	0.200	0.10	0.50
**3**	0.200	0.40	2.00
**4**	0.200	0.60	3.00
**5**	0.200	1.00	5.00
**6**	0.200	5.00	25.00
**7**	0.020	0.40	20.00
**8**	0.040	0.40	10.00
**9**	0.080	0.40	5.00
**10**	0.133	0.40	3.00
**11**	0.166	0.40	2.40
**12**	0.200	0.40	2.00
**13**	0.240	0.40	1.67
**14**	0.400	0.40	1.00
**15**	0.800	0.40	0.50
**16**	0.133	0.40	3.00
**17**	0.166	0.50	3.00
**18**	0.200	0.60	3.00
**19**	0.166	0.40	2.40
**20**	0.166	0.50	3.00
**21**	0.166	0.60	3.60

**Table 3:** Slope values of the straight lines fitted to the experimental data of Figure 3 in the interval 0–30 Gy, indicating the sensitivity to the radiation dose of the set of samples prepared with different FAS concentrations. The coefficients of determination are also reported. Uncertainties correspond to one standard deviation.

(FAS) mM	Slope (Gy^−1^)	R^2^
**5.00**	6.99 ± 0.05	0.9997
**1.00**	7.78 ± 0.06	0.9997
**0.60**	7.98 ± 0.06	0.9998
**0.40**	8.22 ± 0.07	0.9996

**Table 4:** Slope values of the straight lines fitted to the experimental data of Figure 6, indicating the sensitivity to the radiation dose of the set of samples prepared with different XO concentrations. The dose interval considered for the fitting procedure and the coefficients of determination are also reported. Uncertainties correspond to one standard deviation.

(XO) mM	Slope (Gy^−1^)	Linear Dose Interval (Gy)	R^2^
**0.800**	4.97 ± 0.02	0–42	0.9999
**0.400**	6.52 ± 0.04	0–35	0.9998
**0.240**	7.88 ± 0.06	0–28	0.9997
**0.200**	8.22 ± 0.06	0–28	0.9998
**0.166**	8.37 ± 0.11	0–28	0.9991
**0.133**	8.78 ± 0.05	0–21	0.9999
**0.080**	9.07 ± 0.38	0–14	0.9966

## Data Availability

Not applicable.
